# Adaptation of convolutional neural networks for real-time abdominal ultrasound interpretation

**DOI:** 10.3389/frai.2025.1718503

**Published:** 2025-12-09

**Authors:** Austin J. Ruiz, Sofía I. Hernández Torres, Eric J. Snider

**Affiliations:** 1 Organ Support and Automation Technologies Group, U.S. Army Institute of Surgical Research, JBSA Fort Sam Houston, San Antonio, TX, United States; 2 Department of Surgery, Long School Medicine, UT Health San Antonio, San Antonio, TX, United States

**Keywords:** point of care ultrasound, deep learning, convolutional neural network, triage, abdominal hemorrhage, diagnostics

## Abstract

Point of care ultrasound (POCUS) is commonly used for diagnostic triage of internal injuries in both civilian and military trauma. In resource constrained environments, such as mass-casualty situations on the battlefield, POCUS allows medical providers to rapidly and noninvasively assess for free fluid or hemorrhage induced by trauma. A major disadvantage of POCUS diagnostics is the skill threshold needed to acquire and interpret ultrasound scans. For this purpose, AI has been shown to be an effective tool to aid the caregiver when interpreting medical imaging. Here, we focus on sophisticated AI training methodologies to improve the blind, real-time diagnostic accuracy of AI models for detection of hemorrhage in two major abdominal scan sites. In this work, we used a retrospective dataset of over 60,000 swine ultrasound images to train binary classification models exploring frame-pooling methods using the backbone of a pre-existing model architecture to handle multi-channel inputs for detecting free fluid in the pelvic and right-upper-quadrant regions. Earlier classifications models had achieved 0.59 and 0.70 accuracy metrics in blind predictions, respectively. After implementing this novel training technique, performance accuracy improved to over 0.90 for both scan sites. These are promising results demonstrating a significant diagnostic improvement which encourages further optimization to achieve similar results using clinical data. Furthermore, these results show how AI-informed diagnostics can offload cognitive burden in situations where casualties may benefit from rapid triage decision making.

## 1 Introduction

Point of care ultrasound (POCUS) is commonly used for evaluating internal, trauma-based injuries providing real-time diagnostics (Gleeson and Blehar, 2018; Theophanous et al., 2024). During emergency triage efforts, having a non-invasive, deployable imaging tool, such as POCUS, in a pre-hospital setting can be leveraged to better provide urgent treatment to the most severe casualties, ultimately reducing preventable trauma deaths (Dubecq et al., 2021). Despite the advantages of POCUS and ongoing advancements with the technology, the effective deployment of POCUS for triage ultimately depends on the operator’s ability to interpret US images and classify injury. Specifically for military situations, there is an expected shortage of medical providers in combat casualty care, especially for mass casualty situations (Townsend and Lasher, 2018). As such, the true benefit of POCUS for diagnosing and triaging abdominal injuries cannot be fully realized with limited trained sonographers readily available. This challenge extends to the civilian emergency medicine setting, as rural, remote locations would benefit from improved triage imaging tools when less specialized personnel and resources are available and definitive emergency care may be delayed (Russell and Crawford, 2013). We postulate that artificial intelligence (AI) can be leveraged to interpret US captures to classify positive and negative hemorrhage injuries in the abdomen. For this purpose, the POCUS procedure of choice is the Focused Assessment with Sonography for Trauma (FAST) exam where the pericardium and abdomen are evaluated for free fluid in spaces around the kidneys – left and right upper quadrant (LUQ, RUQ) and pelvic or bladder (BLD) regions (Scalea et al., 1999).

In the medical imaging space, AI models have been developed to analyze medical imaging data and provide diagnoses of several abdominal and pelvic pathophysiologies, including traumatic abdominal and pelvic injuries (Cai and Pfob, 2025). For example, Leo et al. (2023) have developed a real-time object detection model for the identification of free fluid at the RUQ scan site using US FAST exam images collected from 94 patients. This study indicated that its motivation for developing a hemorrhage detection model at the RUQ site was due to the studied trend of fluid accumulation in the abdominal region first appearing at the RUQ scan site (Lobo et al., 2017). The 5-fold cross-validation study achieved a YOLOv3 model performance of 0.95 sensitivity, 0.94 specificity, 0.95 accuracy, and 0.97 AUC and IOU hemorrhage detection scores of 0.56 (Leo et al., 2023). Furthermore, Cheng et al. developed a deep learning (DL) model using the ResNet50-V2 architecture and images from 324 patients toward identification of free-fluid in Morrison’s pouch at the RUQ scan site with performances of 0.97 accuracy, 0.985 sensitivity, and 0.913 specificity on the test set (Cheng et al., 2021). Another study related to DL applications for these medical imaging scan sites is Kornblith et al. (2022) development of classification models for identifying scan sites using 4,925 FAST exam videos and approximately 1 million US images acquired from 699 pediatric patients achieving 0.952 accuracy and 0.96 accuracy for correct identification of the RUQ and BLD scan sites, respectively, using a ResNet-152 model. This study provides an overview of the model’s ability to classify the scan sites, not an injury diagnosis classification. Additionally, a dataset of 2,985 US images collected from patients with abdominal free fluid were used to develop a classification model for the severity of free fluid (indicated as Acites-1, Ascites-2, and Ascites-3). A U-net model was able to achieve sensitivity and specificity ranging from 0.944–0.971 and 0.681–0.863 for Ascites-1 and Ascites-2, respectively (Lin et al., 2022). However, it was noted that this classification model was applied to images of the abdominal cavity including the liver and spleen in view but did not include images with the bladder in view.

Previously, our research team developed DL models for classifying injuries in the abdominal and thoracic regions using data captured from animal experiments (Hernandez Torres et al., 2024, 2025). While training data subsets were able to achieve accuracies of 0.62 and 0.79 for the BLD and RUQ scan sites, a later real-time (RT) performance evaluation showed that accuracy dropped to 0.59 and 0.70 in each respective scan site. This showed that despite initial accuracy, the models still struggled to generalize enough to effectively classify injury status when implemented in an RT experiment. In a more recent study, a focused standardized approach to preprocess data and fine tune model architecture parameters for thoracic scan sites was implemented with success of reaching a target accuracy of approximately 0.85 (Ruiz et al., 2025). Due to findings from this RT experiment, the objectives of this brief report are:

- Use retrospective swine dataset of abdominal scan points to explore new AI training modalities.
- Use a frame-pooling approach for classification of the ultrasound scans to add injury context, enhancing AI model performance for injury diagnosis
- To validate new deep learning architecture methods with data from RT animal experiments, retrospectively benchmarking model performance in a RT setting.

## 2 Materials and methods

### 2.1 Data capture and curation

Ultrasound scans used for AI model training in this study were collected from multiple approved swine research protocols. Research was conducted in compliance with the Animal Welfare Act, implementing Animal Welfare regulations, and the principles of the Guide for the Care and Use for Laboratory Animals. The Institutional Animal Care and Use Committee at the United States Army Institute of Surgical Research approved all research conducted in this study. The facility where this research was conducted is fully accredited by the AAALAC International. Live animal subjects were maintained under a surgical plane of anesthesia and analgesia throughout the studies. Abdominal ultrasound scans from the right upper quadrant (RUQ) and pelvic (BLD) abdominal regions were used as the primary datasets for the purposes of this study.

**Figure 1:**
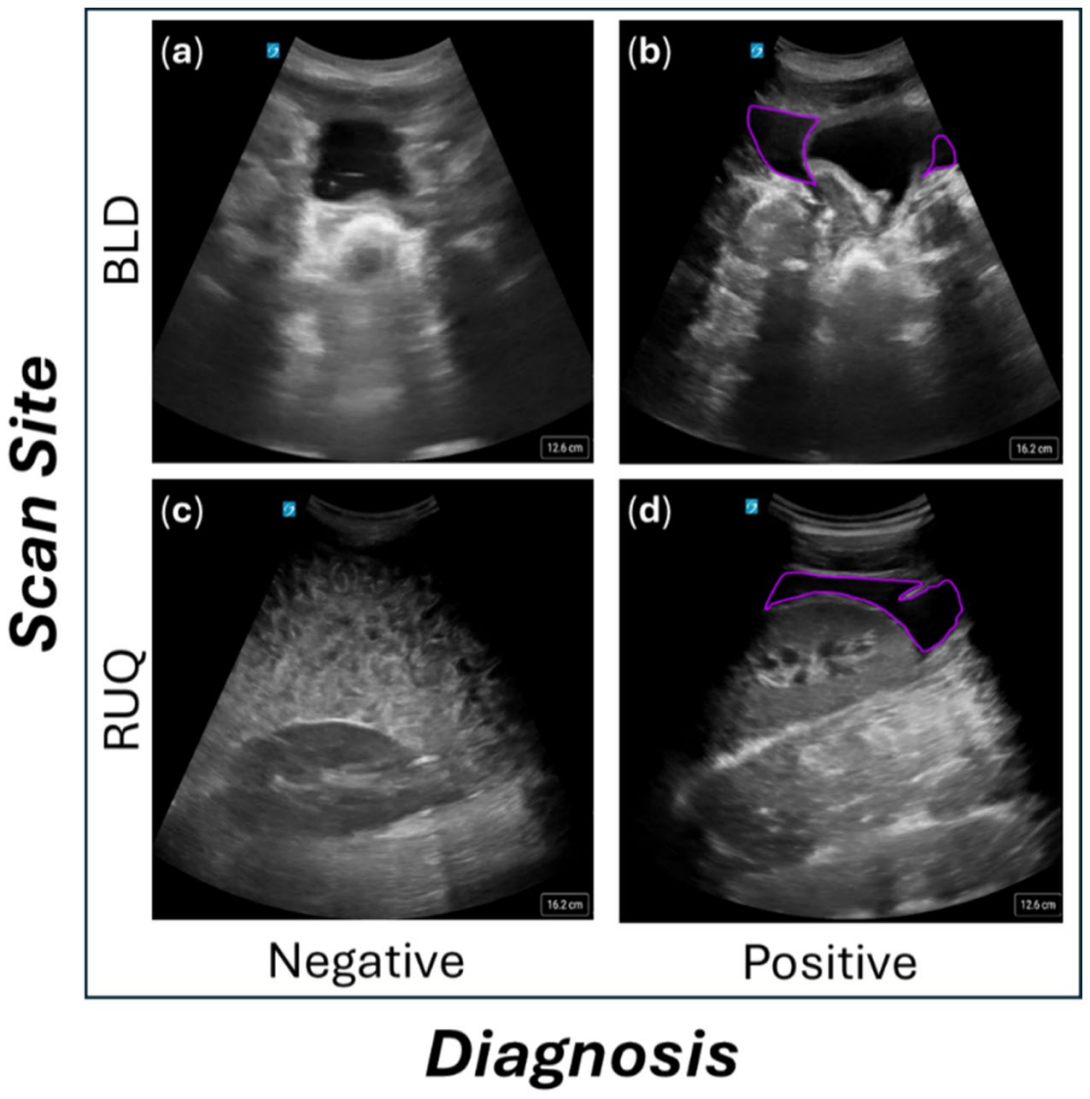
Representative ultrasound scan frames from the abdominal scan sites. Images on the top row are from the BLD scan site for negative **(a)** and positive **(b)** injury classifications. Images on the bottom row are from the RUQ scan site for negative **(c)** and positive **(d)** injury classifications. US images with injury were annotated with a purple perimeter around the fluid, located around the left and right side of the bladder for BLD and above the kidney for RUQ.

Scans were exported from a Sonosite PX Ultrasound System (Fujifilm, Bothwell, WA) as 30-s videos at the native frequency of 30 frames per second (FPS). Exported US captures were labeled as positive or negative for injury (presence or absence of free fluid, respectively) and grouped by scan site, subject ID, and injury. The data were further curated by the research team as described previously (Hernandez Torres et al., 2025) to score image quality, injury severity (for positive cases), and transducer steadiness during video capture. For image quality, the research team qualitatively scored the clarity of the anatomical features between 1 (best) and 5 (worst); quality was reduced by any appearance of shadows in the image and probe contact with skin. Images taken after the swine subjects were positive for injury were sub-stratified between obvious, large accumulations of fluid vs. slight, small fluid pockets. Example frames from the ultrasound dataset are shown for the scan sites of focus and at each injury state on Figure 1. For the last three subjects, images were captured using both the US system and recorded to a Windows computer to use as blind testing and as representative real-time data, respectively. For these test subjects, the same number of video clips were recorded pre- and post-injury, resulting in an even distribution of data.

### 2.2 Advanced image processing

Our team’s previous training pipelines implemented preprocessing techniques focused on normalized measurements of pixel intensity metrics such as brightness, contrast, and a textural metric kurtosis (the skewness of the images distribution of pixel values) (Ruiz et al., 2025). These metrics were used to develop a confidence interval-based filter which improved several performance metrics such as sensitivity and F1 scores. These preprocessing methods were initially applied to the BLD scan site for initial performance evaluation; however, they were not applied to the RUQ scan site due to success achieved from other methods. These preprocessing methods were also not combined with “frame-pooling” methods mentioned below, given results reached target accuracy without them.

### 2.3 Deep learning model training

Once the image metric preprocessing method was evaluated for the BLD scan site, the US captures were set up for training. Using labels for groups of subjects representing different data capture experiments (as described in the previous study), data were configured into groups of subjects. For loading pre-existing models, tensor augmentations, and data loading the PyTorch framework (ver. 2.2.0) was used in a Python environment (ver. 3.11.7) to script training and evaluation tools. Starting with model architecture choice, the team of researchers compared two lightweight model architectures MobileNetV3 (Howard et al., 2017, 2019) and EfficientNet-B0 (Tan and Le, 2020), due to their computational efficiency and accuracy optimization strategies. After evaluating the results and finding better performance with the EfficientNet-B0 architecture (data not shown), we further implemented methods in EfficientNet-B0 architecture and evaluated a larger version of the architecture, EfficientNet-B2 to train and evaluate model performance with more model parameters and amplified channel layers to test for increased performance.

Across training models on each model architecture, three groups were labeled from the total list of subjects captured, where each group was left out of training and validation for the data loaders used in training. The combined data for the remaining groups used an 80–20% split for training and validation, by subject, respectively. The chosen hyperparameters for training included a batch size of 32, Adaptive Moment Estimation algorithm for optimization (Kingma and Ba, 2017), early stopping function of 10 epochs, learning rate of 0.001, and a maximum of 100 epochs. These hyperparameters were chosen as a starting point from results that the team previously observed when comparing models and hyperparameters in a more exhaustive approach for different scan sites. Initially, pre-trained weights from ImageNet were used for training BLD DL models, however, results were poor, indicating the model’s inability to learn the appropriate features. Because of this, the research team abandoned the approach to use pre-trained weights in favor of using weights trained from scratch, with Kaiming Initialization, a commonly practiced method for initializing weights at random values that allowed for a stable variance when choosing random integers for input so the weights do not vanish or explode (pytorch, n.d.). The max count for epochs was increased to 200 to allow the model to train longer with the weights initialized.

After observing data imbalance in the groups of subjects, a k-fold cross-validation approach was implemented, where a stratified representation of data captures from previous studies was grouped for training and validation. For BLD scan site, 29 videos (26,100 images) were used for training and validation, and 8 US videos (7,200 images) were left out for testing, which accounts for roughly 20% of the total data for the scan site. With the RUQ scan site’s dataset, 33 videos (38,574 images) were used in training and validation, while 13 videos (25,800 images) were used for testing, resulting in 28% of the RUQ dataset used for testing.

**Figure 2:**
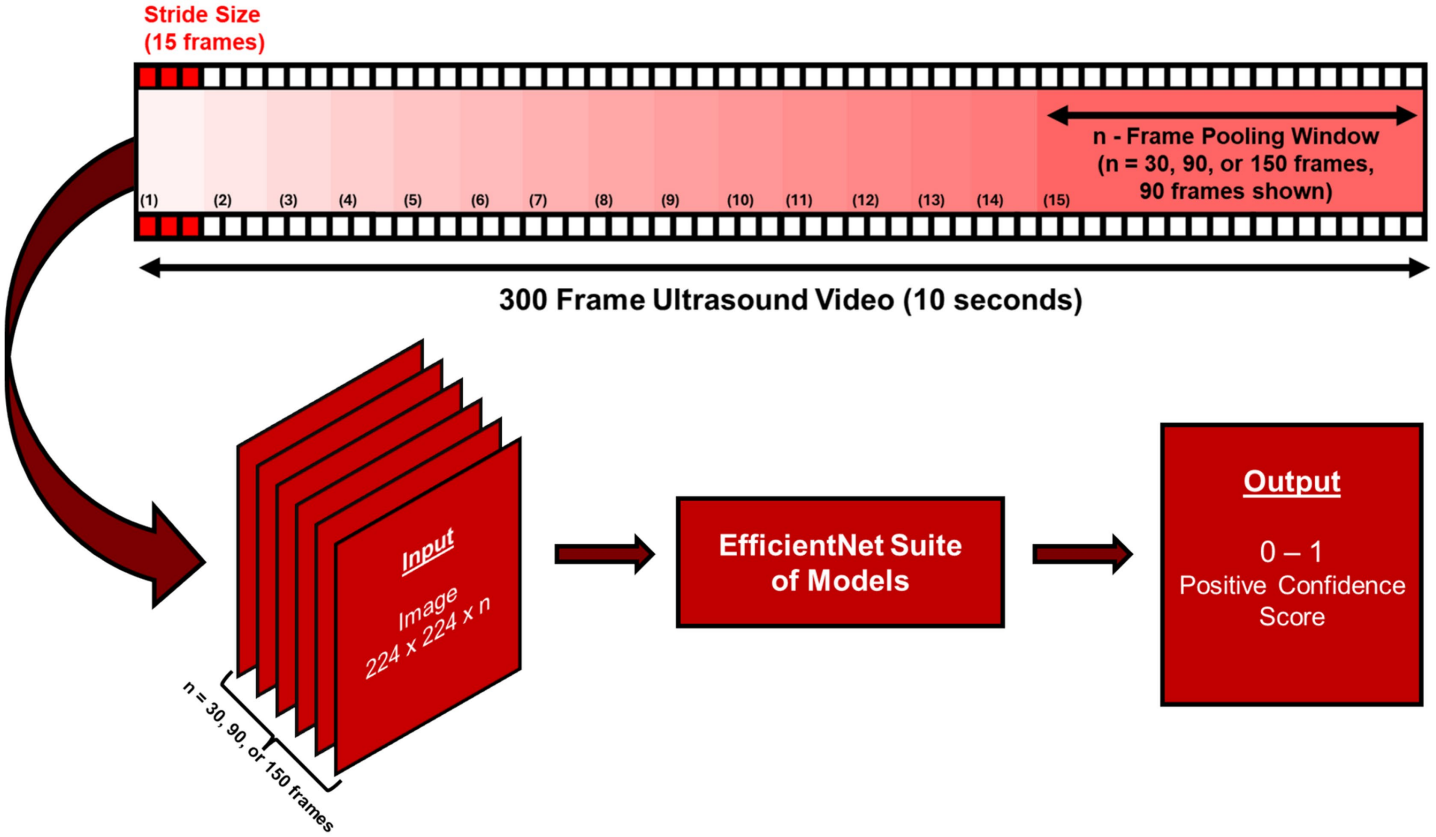
Overview of the frame pooling methodology for multi-channel model training. An *n* number of frames were pooled from each video with a 15-frame stride during training across the entire video. These pooled frames were input to EfficientNet-B0 or -B2 and a binary score in terms of a confidence score for each prediction were output.

After stratifying classifications for loading the dataset splits and using weights initialized from scratch, the models’ performances in the testing set were still low. Due to this, we wanted to explore a different way to load input tensors for the convolutional neural network to account for multiple seconds worth of frames. This way, the presence of fluid taken at multiple angles of the US scan can be accounted by the model for each input. By patching the first convolutional layers in the model architecture to change the number of channels that the model was able to process, the model backbone was customized to handle multi-image inputs. To “pool” images together in sequences as they were exported from the US capture, in addition to the labels for the dataset, a new label for identifying which video each image belonged to was used. Images from each video were pooled consecutively at different video segment sizes, with a stride of 15 images (Figure 2). Different channel width or window sizes for the images were tested in training to compare differences between collecting frames for shorter and longer durations of US capture. With a stride of 15 images, an overlap for each window was collected for the duration of the video, with padding so each window or sequence of images maintained the same number of images in them before being batched together and loaded as input tensors. The hyperparameters chosen for this instance of training remained the same as the initial setup, with pre-trained weights and a max epoch count of 100.

The chosen augmentations for each tensor throughout every iteration of model training included PyTorch’s RandomResizedRecrop method, which chooses a crop of a randomly selected amount between 80 and 100%, a random aspect ratio to resize to between a variation of 3:4 and 4:3, and resizing after. Another PyTorch augmentation method implemented was ColorJitter, which randomly adjusts contrast, brightness and saturation based on a range of percentages for the initial metric values. The chosen ranges for these metric adjustments were ± 20%, ± 10%, ± 10%, for brightness contrast and saturation, respectively. Lastly, an augmentation that uses 50% probability that the image would be flipped horizontally was implemented.

### 2.4 Model assessment and performance evaluation

In addition to the test evaluation splits from the training script, a separate tool was developed to load and inference the trained models for making predictions on real time (RT) captured data. The RT captured data from the three additional, totaled 54 streams of videos for the BLD scan site and 43 streams of videos for the RUQ scan site. Just like in the training methodology, captures were pooled into 150-channel windows at 30 s strides to decrease prediction output time. A prediction was made on each collected window for each video, followed by an average of the *softmax* output confidence scores for each class, wherein a total prediction on the video was labeled on the average of the confidence scores to give a final prediction on the processed RT video. For showing the improvement of frame-pooling on overall test accuracy, statistical analyses using a McNemar test (NCSS 2025 Statistical Software Application) were used to compare 150-channel windows to the single channel for both RUQ and BLD results. Results for RT and holdout test were compiled for when both models were correct, incorrect, or only one model was correct, for input to the paired McNemar test. *p*-values less than 0.05 indicated statistically significant differences between the model pairs and results for these tests are described in the text when applicable.

Overall model performance was assessed using conventional performance metrics – accuracy, precision, sensitivity and F1 scores – for both the holdout test data and RT test data for both RUQ and BLD. Receiver operating characteristic curves (ROCs) were constructed for a range of confidence thresholds for binary predictions and area under ROC (AUC) was quantified. In addition, each performance metric was evaluated for a range of confidence thresholds to characterize the optimal confidence interval for these DL model types in both RT testing and holdout testing. For the optimal threshold, confusion matrices were constructed both holdout and RT test data to summarize the distribution of prediction for both RUQ and BLD.

## 3 Results

### 3.1 Model performance on the pelvic or bladder scan site

**Figure 3:**
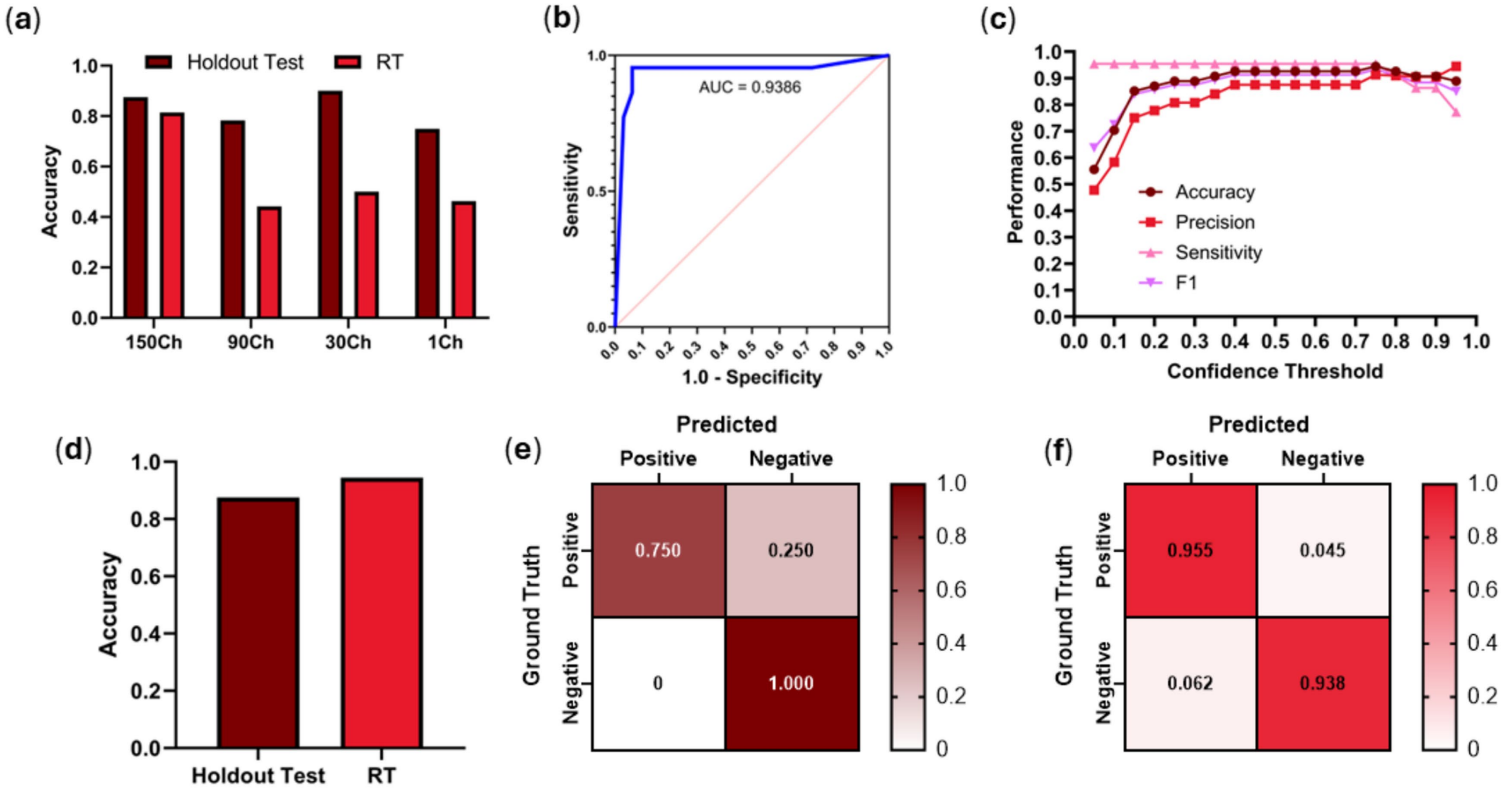
BLD model performance using frame-pooling input and different prediction confidence thresholds. **(a)** Comparison of holdout and real-time test results for different frame pooling amounts using EfficientNet-B0 architecture. **(b)** ROC curve using EfficientNet-B2 architecture. **(c)** Comparison of accuracy, precision, sensitivity and F1 metrics for different confidence thresholds using EfficientNet-B2 architecture. **(d)** Comparison of holdout vs. RT testing dataset accuracies when evaluated on a confidence threshold of 0.75 using EfficientNet-B2 architecture. Confusion matrices for EfficientNet-B2 (0.75 confidence threshold) for **(e)** holdout test and **(f)** RT results. Results for each confusion matrix are normalized to ground truth positive and negative counts.

Initial model evaluation results using EfficientNet-B0 before changing the backbone for the model to patch the input size for channel widths on the first convolutional layer had a holdout testing accuracy of 0.70 and a RT evaluation score of 0.40, which illustrates the model’s inability to generalize on completely blind subjects (Figure 3a). Using frame-pooling input layers, evaluation showed window sizes of 30 frames and 90 frames performed worse than 150 frames for RT predictions, with accuracies of 0.90 (test) and 0.50 (RT) for a 30-frame width, 0.78 (test) and 0.44 (RT) for a 90-frame width, and finally 0.88 (test) and 0.81 (RT) for a 150-frame width (Figure 3a). As such, the 150-frame pooling size was selected for the BLD scan site. Comparing 150-frame pooling vs. a single frame, there was a significant difference in performance for RT test data (*p* = 0.0003, *n* = 54 videos), but it was not significant for the holdout test data (*p* = 0.5637, *n* = 8 videos).

Using the EfficientNet-B2 architecture, AUC was 0.9386 with RT predictions, indicating a strong RT prediction performance (Figure 3b). The optimal confidence was identified as 0.75 based on the ROC curve and the best balance of performance metric scores across different confidence values (Figure 3c). This was determined using RT datasets, but similar confidence thresholds were optimal for holdout test data (data not shown). At the optimum confidence threshold of 0.75, accuracy (0.94), precision (0.91), sensitivity (0.95), and F1 score (0.93) were much improved over previous work which had a RT accuracy of 0.59 for the BLD scan site. To validate the optimal confidence value, the best confidence threshold of 0.75, resulted in an accuracy of 0.88 with holdout test US images and an accuracy of 0.94 for RT data (Figures 3d–f). These results show an increase of 0.35 from initial model performance from previous experiments’ models.

### 3.2 Model performance on the right upper quadrant scan site

**Figure 4:**
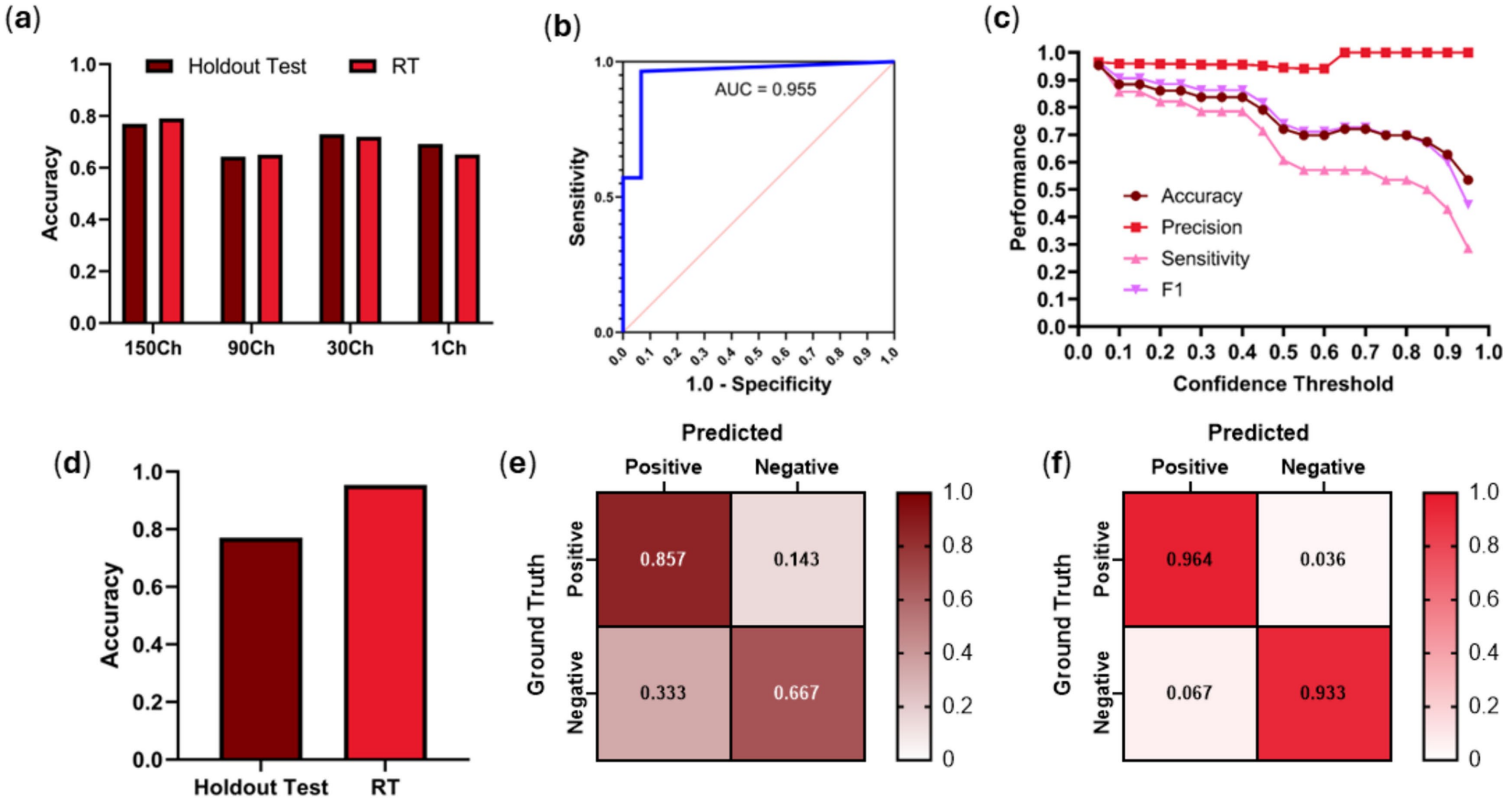
RUQ model performance using frame-pooling input and different prediction confidence thresholds. **(a)** Comparison of holdout and real-time test results for different frame pooling amounts using EfficientNet-B0 architecture. **(b)** ROC curve using EfficientNet-B2 architecture. **(c)** Comparison of accuracy, precision, sensitivity and F1 metrics for different confidence thresholds using EfficientNet-B2 architecture. **(d)** Comparison of holdout vs. RT testing dataset accuracies when evaluated on a confidence threshold of 0.05 using EfficientNet-B2 architecture. Confusion matrices for EfficientNet-B2 (0.05 confidence threshold) for **(e)** holdout test and **(f)** RT results. Results for each confusion matrix are normalized to ground truth positive and negative counts.

A similar training approach was used for the RUQ scan site wherein we first assessed optimal input frame pooling approaches using EfficientNet-B0. Single frame predictions had a RT prediction accuracy of 0.651 while the frame pooling approaches had accuracies of 0.720, 0.650, and 0.791 for 30-, 90-, and 150-channels, respectively (Figure 4a); 150-channel frame-pooling was selected as the optimal image input approach. However, comparing 150-frame pooling vs. a single frame, there were no significant differences in performance for RT test data (*p* = 0.1083, *n* = 43 videos) and holdout test data (*p* = 0.5637, *n* = 13 videos).

With EfficientNet-B2 and the 150-frame input layer, strong ROC performance was evidenced by an AUC of 0.955 (Figure 4b). Of interest, the optimal confidence threshold was heavily biased towards positive injury predictions at 0.05, meaning if the model had even a suspicion of being positive for injury that would be the prediction (Figure 4c). This was the case for both test holdout and RT test datasets. At this threshold, accuracy (0.953), precision (0.964), sensitivity (0.964), and F1 score (0.964) all scored high for predicting free fluid around the kidneys in the RUQ view. Using the best performing confidence threshold from the holdout test set of 0.05, an accuracy performance was 0.77 for holdout test data while stronger accuracy performance was evident at 0.95 for the RT test set (Figures 4d–f). This is a 25-point improvement over previous work RT accuracy of 0.70, highlighting the benefit of the frame pooling methodology.

## 4 Discussion

The main purpose of this study was to develop injury classification models for BLD and RUQ scan sites of an eFAST examination as previous blind model performance was below 70% accuracy in RT predictions (Hernandez Torres et al., 2025). DL models for binary classification of injury states have the potential to automate this medical imaging diagnosis and simplify triage in the pre-hospital military and civilian care method if overall model performance is improved. DL models that show promising signs of effectiveness for this application, can be scaled and transfer-learned to provide a potential life-saving solution for trauma casualties in austere environments. Despite previous efforts, the BLD and RUQ scan site struggled to adequately determine classification of streamed data capture in a recent animal experiment. The utility of AI is measured only by its ability to reliably perform in a RT scenario, rapidly identifying presence of injury.

The challenges that the DL models must overcome to generalize well enough to reliably predict positive or negative for injury states are unique to the two abdominal scan sites mentioned throughout this study. Starting with BLD, the underlying bladder organ anatomy and its ability to take different shape and form poses challenges for the DL models to identify the correct features. For example, the bladder can vary in the amount of urine it contains before injury, making the shape variable in shape and size. Additionally, there are several other organs and structures that can make it difficult to interpret for injury around the bladder such as the bowel, uterus, ovaries or prostate. The variability that these structures can introduce could potentially be misinterpreted as artifacts.

Aside from anatomy, the variability of the injury itself poses difficulties when attempting to classify between uninjured and injured states. In real-world scenarios, this gets further confounded by the type of trauma (blunt or penetrating) causing the injury, and where the fluid is coming from. In the case of penetrating trauma, a gunshot can introduce debris that will be difficult to identify if the model does not have enough data for training. In blunt trauma, reported cases for patients that got injured from a motor vehicle, or for deceleration will also influence where and how severe the injury looks. Combining this with the heterogeneity of data captures at these scan sites can give DL models trouble in learning the appropriate features. This highlights the importance of adding clinically relevant, human images to training datasets before deploying diagnostic algorithms such as the ones trained in this work. However, the same data processing and sampling during training can be applied to the clinically relevant dataset to produce more accurate models as shown by the work here.

Introducing multi-channel frame-pooling to the model’s architecture resulted in a considerable amount of improvement over previous methods. Originally, features were only extracted and trained at a single image level, which may look different depending on time of the scan. This is due to what regions of the capture were present, which heavily depended on the angle of data capture at the given fidelity of 30 FPS. By implementing a rolling window of sequentially captured images and summing the convolutional layers, the input multichannel images that the model was using to learn is more representative of how a medical provider may interpret US results. In practice, trained experts evaluate the US and provide a diagnosis based on several frames, not a single US image, in order to use context of the multiple angles of data capture before making a diagnosis. This approach makes sense from a medical perspective with the presence of several anatomical features in the abdominal region. Additionally, with initial strong results from EfficientNet-B0, we observed even stronger results after retraining models with EfficientNet-B2. This is likely due to its larger model architecture compared to EfficientNet-B0 containing more trainable parameters (9.1 M vs. 5.3 M parameters) and more channels to capture subtle signal differences from input of stacked US frames. This could imply that even larger configurations of the EfficientNet suite of model architectures could improve performance even further but that could come at an increased likelihood of model overfit.

After switching model architectures from EfficientNet-B0 to EfficientNet-B2 and using the training holdout set to choose a confidence threshold for positive predictions and implementing it in the retrospective RT data capture pipeline, average accuracies for both scan sites achieved over 0.93. To achieve these results, optimal confidence thresholds for predictions varied greatly between BLD (0.75 confidence threshold) and RUQ (0.05 confidence threshold). The underlying reason between different thresholds is unknown, but these values were optimal to avoid false positive and negative results. They will need to continue to be fine-tuned with different datasets and scan locations going forward as the optimal selection varied so greatly between these two use cases. While these results are significantly higher than the previous experiment’s RT evaluation, they were extrapolated from videos captured of only three swine subjects. Given how variable and heterogeneous the features of clinical examples of US injuries are at the RUQ and BLD scan site, the limitation of the lack of specificity for specific types of traumas that may be underrepresented in the evaluation pipeline data needs to be considered. Ultimately, to truly benchmark model performance, the models need to be inferenced in a live RT setting.

Aside from limitations of the dataset itself, there were no further investigations in fine-tuning model hyperparameters for further improving the CNN models. Another limitation for this study is that the CNN model architecture is being repurposed to handle multiple US images in batches with randomized weights, meaning the kernel that performs convolution applies to a number of US images at once, rather than performing convolution on each image separately before comparing them. Recurrent Neural Networks (RNNs) are designed to take sequential data to handle maintenance of previous inputs at a single input level (Schmidt, 2019), which performs a function that translates to temporal “awareness” more natively than the current approach. As such, the next steps for this study may include the development of RNNs such as a Long-Short-Term-Memory (LSTM) Network that uses recurrent connections in the architecture built for processing sequential data. With further exploration and improvements to these DL models, the same methods can be applied for other scan sites, such as the Left Upper Quadrant (LUQ) and the Pericardial (PC) view.

## Data Availability

The data analyzed in this study is subject to the following licenses/restrictions: The data presented in this study are not publicly available because they have been collected and maintained in a government-controlled database located at the U.S. Army Institute of Surgical Research. This data can be made available through the development of a Cooperative Research and Development Agreement (CRADA) with the corresponding author. Requests to access these datasets should be directed to Eric J. Snider, eric.j.snider.civ@health.mil.
